# Abstracts from Foreign Journals

**Published:** 1900-07

**Authors:** 


### ABSTRACTS FROM FOREIGN JOURNALS.

Dourine and its Parasite. In the *Recueil de Médecine Vétérinaire* has appeared an article of great value and interest by G. Schneider and M. Buffard (the former an army surgeon, in charge of the Military Hospital at Oran, Algeria, the latter an army veterinarian), begun in the February number, and concluded in April. In the year 1896, Rouget discovered in the blood of a stallion with dourine a parasite of the genus Trypanosoma. He made an excellent study of this organism, which belongs to the family Cercomonas. He inoculated rats, rabbits, and dogs with the parasite, and recognized the striking analogy between the symptoms produced in the latter animal and those of dourine. The accidental loss of the parasite prevented Rouget from inoculating horses, and determining its specific character.

Schneider and Buffard have found again the parasite of Rouget, and have been able to prove its specificity. They review the classic picture of dourine as seen in the horse, the mare, the ass, and the she-ass, and add observations of their own, chiefly as to the appearance of cutaneous plaques. These appear forty, forty-five, and sixty days after the infecting coitus; they are usually unstable, and simple exercise may cause them to disappear. Sometimes they persist for five to eight days. Their appearance may be preceded by an eruption of buttons, which sometimes exude a serous fluid which glues the hair in masses. Treatment with quinine sulphate was tried, but though the sick animals seemed to improve for some days, the disease was not checked in its fatal course.

The authors found the Trypanosoma in the blood of two stallions and two donkeys. The character of this parasite is analogous to that producing "surra" and "nagana." It is easily stained by the methods commonly employed in bacteriology, but it has not been possible to cultivate it. It is not always easy to discover the parasite in the sick animal. It is found in blood taken about the seat of the engorgements and cutaneous plaques, and especially just at the time of their first appearance. According to the authors the formation of these plaques is due to the plugging up of the minute vessels by masses of the Trypanosoma.

The blood (even when it seems to contain no parasites), the semen, the vaginal mucus, and the softened portions of the cord, are virulent. Experimental inoculation is possible in the horse, ass, dog, rabbit, rat, and mouse; subcutaneous inoculation being the best method of procedure. Infection is also possible by coitus, intra-cranial inoculation, and by rubbing the virulent matter into the mucous membrane of the vagina or the conjunctiva. According to the resistance of the animal, and the number of parasites introduced, we obtain, especially in the dog, more or less grave manifestation of the disease. Nocard showed, in 1892, that intra-ocular inoculation of material taken from one of the softened areas in the cord of a horse with dourine, provoked in dogs a disease closely resembling dourine in the horse.

Schneider and Buffard have been able to bring about the same result by inoculation with the blood as well as the cord. They recognize four stages in the development of the experimental disease in dogs: First, incubation; second, œdema at point of inoculation, with heat and pain; œdematous infiltration at back part of abdomen and in genitals; balanitis in dogs; vaginitis and sometimes abortion in bitches; continued fever; third, emaciation, disorders of locomotion, the formation of cutaneous plaques, arthritis, purulent conjunctivitis, ulcerative keratitis. Lastly, in the fourth stage, there is extreme cachexia, with paralysis or death by syncope. Usually the evolution of the disease occupies a number of weeks.

In the horse and mare the authors find that between the spontaneous and experimental disease there is no difference either in the symptoms or in the lesions.

The morphology and evolution of the Trypanosoma was studied principally in blood taken from the cutaneous plaques. The parasite disappears very quickly from the blood of animals.

The authors give a detailed description of the various phases of the disease as it occurred spontaneously in an ass and a horse, and of the experimental disease in three horses. They succeeded in transmitting dourine from the horse to the dog, and *vice versa*. They also communicated the disease experimentally from the horse to the mare. They proved that a dog could contract dourine by coitus with a bitch experimentally infected, and that the rabbit may be infected in the same way.

Schneider and Buffard say that the affection produced in this way was unquestionably dourine. They have considered well the possibility that the disease they were studying might be "surra," and not dourine, the two diseases having certain points of resemblance, and both being caused by a Trypanosoma. They conclude that in both the spontaneous and experimental cases studied by them the disease was "absolutely differentiated from surra by the feeble thermic reaction, the engorgement of the genitals, the formation of cutaneous plaques, and the preservation of the appetite until death. In surra, on the contrary, there are intense fever, œdema of the belly and especially of the extremities, absence of plaques, and absolute loss of appetite. The mode of propagation by coitus is characteristic of dourine, while surra has an epidemic character, and has never been observed in Algeria."

Schneider and Buffard cannot believe that the Trypanosoma is an accidental and harmless organism in the disease. During five months they examined the blood of many animals—mice, rats, horses and stallions, dogs and fowls—with various diseases, chronic and incurable, without ever finding the Trypanosoma. They have also considered the possibility that they were dealing with cases of dourine, in which the Trypanosoma occurred as a complication, or in association with dourine. This objection they are able to rule out also, and conclude that the Trypanosoma described by them is the true and specific parasite in dourine.

Besides the great scientific interest of their work, it gives to veterinary medicine two means of diagnosis in doubtful cases—microscopic examination of the blood taken from the cutaneous plaques, and the inoculation of dogs.—*From the Laboratory of the State Live Stock Sanitary Board.*

---

*Bulletin No.* 65 of the Nebraska Experiment Station, prepared by Dr. A. T. Peters, covers the subject of "Blackleg: Its Nature, Cause, and Prevention." A general history of the disease and the value of vaccination is presented in a manner that will arouse the interest and support of stockowners and no doubt lessen materially the losses suffered from this fatal disease. Such work as this, while bringing but a slight direct remuneration to the veterinarian, makes sure and valuable his place and position in every community, and gives a helping hand to a large number of our people whose lot at best is a hard one.

